# Supplementary material for: Association of nasopharyngeal Dolosigranulum pigrum and Corynebacterium species with post-acute sequelae of SARS-CoV-2 in a longitudinal cohort
Source: Microbiol Spectr. 2026 Mar 17;14(4):e02313-25. doi: 10.1128/spectrum.02313-25 (PMC13055391; doi:10.1128/spectrum.02313-25)
Supplement: Supplemental material — Supplemental methods, Table S1, and Fig. S1 and S2. [file spectrum.02313-25-s0001.docx]

**Supplementary Materials S1**

**Beginning of Supplementary Methods**

Sample cohort and storage

The study involved collecting samples from participants enrolled in the HYGIEIA study(13), including 25 healthy controls, 24 patients with influenza, 50 patients with moderate COVID-19 (non-hospitalised/WHO Clinical Progression Scale < 4), and 57 patients with severe COVID-19 (hospitalised/WHO Clinical Progression Scale > 4). Participants were enrolled between August 2020 and March 2024 from Cliniques universitaires Saint-Luc and Grand Hôpital De Charleroi in Belgium. The protocol has received ethical approval from the local ethical committee (2021/30DEC/543) and was registered on ClinicalTrials.gov (NCT05557539). Nasopharyngeal swab samples were obtained from each participant at two time points: first, at the time of diagnosis/study inclusion, and then during a follow-up visit approximately three months later. Around 80% of participants enrolled in the study successfully continued until the second time point (24 healthy controls, 16 influenza patients, 46 moderate COVID patients, and 37 severe COVID patients), with an average follow up time of 81 days between visit 1 and visit 2. NP swabs were flash frozen in liquid nitrogen within 2 hours of sample collection, before being promptly stored in a biobank at -80°C. From this cohort, shotgun metagenomic data were successfully collected for 19, 21, 46, and 48 healthy, influenza, moderate COVID, and severe COVID patients, from visit 1, respectively, and 22, 17, 37, and 11 from visit 2. Participants with both visit 1 and visit 2 data are 16 healthy controls, 15 influenza patients, 36 moderate COVID, and 10 severe COVID. Reasons for unsuccessful data collection included inadequate sample volume and low DNA yield.

To avoid any bias due to sample processing, sample preparation, sequencing and data generation was performed in random batches of 16 samples, plus 1 control sample (Zymo balanced cell control (Catalog No. D6300, Zymo Research, US)) used for quality control purposes. Random batches were created programmatically, ensuring patient sex, cohort group (healthy, flu, mild COVID, severe COVID), sample collection date, and visit number of samples was consistent as regarding the cohort as a whole. Batch effects were investigated by comparing quality-control metrics and control sample composition across batches, but no significant effect size was seen.

Patient clinical data

Clinical data was collected systematically at each study visit by a registered nurse. Additionally, comprehensive clinical histories and patient medical records were obtained via detailed review of electronic medical records by clinical researchers. Collected data encompassed patient demographics, comorbidities, comedications, vaccination status, clinical symptoms (including respiratory, neurological, gastrointestinal, cardiovascular, and general symptoms), COVID severity (graded according to WHO Clinical Progression Scale scores), hospitalization details (admission, discharge, ICU stays), and treatment interventions (oxygen therapy, ventilation support, Extracorporeal Membrane Oxygenation, vasopressors, renal replacement, tracheostomy, and antibiotic, antiviral or immunomodulatory treatments). PASC status at the follow-up visit was assessed by a study nurse and defined as the presence or continuation of symptoms at visit 2 without any obvious alternative explanations.

DNA extraction

DNA extraction was conducted using the ZymoBIOMICS DNA Miniprep kit (Catalog No. D4300, Zymo Research, US), according to the manufacturer instructions with minor modifications. Nasopharyngeal swab samples were removed from the -80°C storage and thawed completely at room temperature. Samples were then vortexed for 1 minute to ensure thorough mixing of the swab and swab media. Under aseptic conditions, the swab tip was carefully cut, using sterilized scissors, into a Zymo BashingBead Lysis Tube containing beads of 0.1-and 0.5-mm sizes. 900 µL of sample media was added to each tube, followed by the addition of 100 µL of DNA/RNA Shield. The tube was tightly capped, vortexed for 5-10 seconds to ensure proper mixing of the contents, and then placed into a Vortex Genie fitted with a 2 mL tube attachment and vortexed at full speed for 45 minutes. Once vortexing was completed, tubes were transferred to a mini-centrifuge and spun at 12,000xg for 1 minute. The ZymoBIOMICS DNA Miniprep kit protocol was then followed from this point forward.

Briefly, 400 µL of supernatant was transferred to a zymo-spin III-F filter and centrifuged at 8,000xg for 1 minute, after which, 1,200 µL of DNA binding buffer was added to the filtrate and mixed via pipetting. 800 µL of mixture was transferred to a zymo-spin IICR column, centrifuged at 10,000xg for 1 minute, and flow through was discarded. This step was repeated twice for a total of 1,600 µL of mixture. The IICR column was then transferred to a new collection tube and washed with 400 µL DNA wash buffer 1, 700 µL DNA wash buffer 2, and 200 µL DNA wash buffer 2, with centrifugation at 10,000xg for 1 minute and discarding of flow through after each addition of wash buffer. DNA was then eluted from the filter into a new tube using 100 µL DNase/RNase free water heated to 60°C and applied directly to the column matrix and centrifuged at 10,000xg for 1 minute. The resulting elute was then reapplied to the column matrix and again, centrifuged at 10,000xg for 1 minute. As a final purification step, the eluted DNA was transferred to a Zymo-Spin III-HRC filter (pre-washed with 600 µL HRC prep solution) and centrifuged at 16,000xg for 3 minutes. After the DNA eluted, a Qubit assay was performed using 2 µL of the purified DNA to quantify the DNA concentration. Purified DNA was stored at -20°C while the remaining lysis tubes were labelled and stored at -80°C for future re-extraction if needed.

Library preparation and sequencing

The Illumina DNA Prep kit (Catalog No. 20018704, Illumina, US) was used for library preparation. To begin, 25-49ng of DNA in a 30 µL volume was added to the well of a 96-well plate, followed by 10 µL bead-linked transposomes and 10 µL of tagmentation buffer 1, and mixed via pipetting. The plate was then placed in a VertiPro thermal cycler (ThermoFisher Scientific, US) at 55°C for 15 minutes. After the run, 10 µL tagment stop buffer was added to each well and mixed via pipetting, before the plate was replaced in the thermal cycler at 37°C for 15 minutes.

Following tagmentation, beads within the wells were then washed by repeating the following procedure three times: the plate was placed on a magnetic stand for 3 minutes until the liquid turned clear and the supernatant was removed, the plate was then removed from the stand and 100 µL tagment wash buffer was added directly to the beads, followed by homogenization through pipetting. After the final homogenization, the plate was placed back on the magnetic stand and the liquid was allowed to clear before the supernatant was removed.

The plate was then removed from the magnetic stand and 40 µL PCR master mix (composed of 20 µL enhanced PCR mix and 20 µL nuclease free water) and 10 µL of a corresponding index adapter (IDT-Ilmn DNA-RNA UD Indexes Set A/B Tagmentation, Illumina, US) were added and mixed via pipetting. Wells of the plate were then sealed and the plate was centrifuged at 280xg for 30 seconds. The plate was placed in the thermo cycler with the following run settings: 68°C for 3 minutes, 98°C for 3 minutes, 5 PCR cycles (98°C 45 seconds, 62°C 30 seconds, 68°C 2 minutes), 68°C for 1 minute, hold at 10°C ∞.

Resulting libraries were then cleaned. The plate was removed from thermocycler and centrifuged at 280xg for 1 minute, then placed on a magnetic stand for 5 minutes to allow the liquid to clear. A total of 45 µL of supernatant was transferred to a clean well on the plate to the right and the plate was removed from the stand. Then, 40 µL of nuclease-free water and 45 µL sample purification beads were added to the new wells and mixed via pipetting. The plate was then incubated at room temperature for 5 minutes, followed by placing the plate on the magnetic stand for 5 minutes to allow liquid to clear. After this time, 125 µL of supernatant was transferred to a clean well on the plate to the right, and the plate was removed from the stand. Next, 15 µL of sample purification beads were added to the new well, mixed via pipetting, and incubated at room temperature for 5 minutes, followed by placing the plate on the magnetic stand for 5 minutes until the liquid was clear. The supernatant was then discarded and beads were washed twice on the magnetic stand using 200 µL 80% ethanol, incubated for 30 seconds each time, before being removed. Finally, the plate was removed from the magnetic stand, and 32 µL of resuspension buffer was added to the beads in each well and pipetted 30 times to resuspend, then incubated at room temperature on a magnetic stand for 2 minutes. A total of 30 µL of supernatant was then transferred to a clean PCR tube.

Following library preparation, concentration and average fragment size of the libraries were then assessed individually, using Qubit and TapeStation, in order to calculate sample molarity. Libraries were then diluted, pooled, and diluted again to a final loading molarity of 750 pM with 2% PhiX spike-in. Finally, sequencing was performed on the Illumina NextSeq 1000 platform using a NextSeq 1000/2000 P2 Flow Cell (17 samples per sequencing run) generating 800M paired-end reads (46M paired-end reads per sample accounting for 2% PhiX spike-in).

Bioinformatics

All sequencing reads were first processed in an Ubuntu environment (v22.04.4 LTS) with a standardized bioinformatic workflow as follows. First, raw paired-end reads were examined for initial quality using FastQC (v0.11.9). To remove adapter sequences and low-quality bases, reads were processed with Trimmomatic (v0.39) using the following parameters: SLIDINGWINDOW:6:20 MINLEN:70.

Next, to eliminate host (human) contamination, trimmed reads were aligned to the human reference genome (GRCh38) using Bowtie2 (v2.4.5). Unaligned reads (i.e., putative non-host reads) were captured in paired FASTQ files and used for downstream analyses. Samples under 500,000 reads remaining were removed (9 participants), remaining samples contained an average of 2 million reads.

Taxonomic classification was then performed on host-depleted reads using Kraken2 (v2.1.3) with a customized database consisting of RefSeq complete bacterial genomes, RefSeq complete archaeal genomes, RefSeq complete viral genomes, and RefSeq complete fungal genomes. These databases were downloaded, alongside the NCBI taxonomy, and the custom database built using the kraken2-build command within Kraken2. Classifications were conducted in paired mode with a minimum of three hit groups and a confidence threshold of 0.05. An average of over 170,000 reads were identified per sample.

To refine taxonomic abundance estimates, Bracken (v2.7) was run on each of the resulting Kraken2 report files for each sample at species level using a high confidence operational taxonomic unit (OTU) read threshold of 200 reads to remove low abundant OTU identifications.

Statistical analysis

Bracken abundance reports were analysed using R (v4.4.1). Prior to analyses, biological contaminants were identified using the “frequency” method from the decontam package (v1.26.0) with a significance threshold of 0.05. Participant samples with fewer than 500,000 total reads were also removed to ensure a minimum read depth across samples. This threshold was selected to remove outlier samples with significantly lower read counts that might bias or otherwise affect downstream analyses. These excluded samples were not included in ANCOM-BC2 or other statistical tests.

Alpha diversity was assessed on a per-sample basis using two complementary indices: observed richness (i.e., the number of taxa present) and Shannon diversity (which accounts for both taxa richness and the evenness of their abundances). Statistical comparisons across patient groups were performed using nonparametric Wilcoxon or Kruskal Wallis tests when appropriate tests. Beta diversity was analysed using Aitchison distance, corresponding to Euclidean distances between centred log-ratio (CLR) transformed abundance values (CLR transformation used a pseudo count of 1). In order to quantify the strength of association between beta diversity and external factors, permutational multivariate analysis of variance (PERMANOVA) was then applied to the CLR data. Alpha and Beta diversity metrics and CLR transformations were processed using functions within the mia (v1.15.36) and vegan (v2.6-8) packages..

Differential abundance was analysed using Analysis of Compositions of Microbiomes with Bias Correction 2 (ANCOM-BC2) (ANCOMBC v2.8.1) with a global prevalence threshold of 10%, a minimum library size of 500 reads, and s0_perc = 0.05. Count data at the species level were used for the analysis, and differentially abundant species were defined as those with a log fold change greater than 1 and a q-value less than 0.05. Relevant confounding factors (e.g., cohort grouping, sex, antibiotic treatment) were incorporated into the model to account for their effects. To further assesses the robustness of differential abundance results, a sensitivity analysis was performed within ANCOM-BC2 to evaluate the impact of potential violations of model assumptions, such as the presence of structural zeros or varying sampling fractions. Sensitivity analysis in ANCOM-BC2 is implemented by re-running the model with alternative pseudo count values. This tests if the estimated log fold changes and significance levels remain stable despite different treatments of zeros in the data, confirming the robustness of the differential abundance results.

To investigate global co-abundance patterns between nasopharyngeal species, we constructed a SparCC-based association network using all available samples (all patient groups, visit 1 and visit 2).

First, we restricted the dataset to prevalent species (mia::subsetByPrevalent). Species were retained if they had a non-zero relative abundance in at least 10% of samples. This step avoids unstable correlations driven by very rare taxa and reduces dimensionality.

SparCC correlations were then computed on the corresponding species-by-sample raw count matrix using the SpiecEasi::sparcc function (SpiecEasi v2.0.1). To assess the statistical support for each correlation, we used the SpiecEasi::sparccboot procedure with 1,000 bootstrap resamples. Two-sided p-values for each species pair were obtained and adjusted for multiple testing using the Benjamini–Hochberg false discovery rate (FDR) procedure across all pairwise correlations.

For network construction, we first set to zero all correlations that did not meet our predefined significance/effect-size criteria (FDR ≥ 0.01 or absolute correlation coefficient |r| < 0.3). These thresholds were chosen to retain only robust, moderate-to-strong associations. The resulting sparse correlation matrix was then converted into a similarity matrix using a signed dissimilarity transformation for non-zero correlations, with dissimilarity set to 1 for pairs with zero correlation (i.e. no edge). This similarity matrix was used to construct an undirected weighted network (igraph v2.0.3). Vertices represent species and edges represent retained co-abundance relationships, with edge weights proportional to the transformed similarity.

For visualisation, we removed isolated nodes (species with no retained edges) and plotted the network using a Fruchterman–Reingold force-directed layout (igraph::layout_with_fr). Edge colours encode the sign of the underlying SparCC correlation (green for positive, red for negative), and edge width is scaled with the magnitude of the correlation. Node size was set proportional to the mean relative abundance of each species across all samples, such that more abundant taxa appear as larger nodes in the network.

**End of Supplementary Methods**

|  | **Cohort disease grouping** | | | | **PASC status** | |
| --- | --- | --- | --- | --- | --- | --- |
|  | *Healthy controls n=25* | *Influenza controls n=24* | *Mild COVID-19 n=50* | *Severe COVID-19 n=57* | *Recovered n=75* | *Developed PASC n=39* |
| ***General demographics*** |  |  |  |  |  |  |
| *Mean Age* | 43 | 58.5 | 44.3 | 63.4 | 47.2 | 55.2 |
| *% Male* | 44 | 54.2 | 38 | 70.2 | 46.7 | 56.4 |
| *% Female* | 56 | 45.8 | 62 | 29.8 | 53.3 | 43.6 |
| *Mean BMI* | 24.6 | 26.8 | 24.7 | 28.6 | 24.8 | 28.3 |
| ***Ethnicity*** |  |  |  |  |  |  |
| *% Caucasion* | 96 | 91.7 | 68 | 71.9 | 76 | 74.4 |
| *% African* | 0 | 4.17 | 8 | 19.3 | 6.67 | 15.4 |
| *% Unknown/Other* | 4 | 4.17 | 24 | 8.77 | 17.3 | 10.3 |
| ***Smoking status*** |  |  |  |  |  |  |
| *% Currently smoking or recently stopped (within 1 year)* | 36 | 45.8 | 22 | 40.4 | 26.7 | 33.3 |
| *Mean pack years of smokers* | 11.1 | 17.5 | 19 | 36.1 | 16 | 21.2 |
| ***Alcohol status*** |  |  |  |  |  |  |
| *% Low use (No alcohol - occasional)* | 80 | 70.8 | 88 | 71.9 | 81.3 | 69.2 |
| *% High use (Regular* | 20 | 25 | 10 | 17.5 | 16 | 23.1 |
| ***% with full round of COVID-19 vaccination prior to inclusion*** | 84 | 81.8 | 90.2 | 64.5 | 87.5 | 61.5 |
| ***Disease severity*** |  |  |  |  |  |  |
| *COVID-19 WHO score* | 0 | 5.08 | 2.49 | 5.51 | 2.42 | 4.44 |
| *% with any disease complications* | 0 | 83.3 | 20 | 93 | 29.3 | 74.4 |
| *% with any active comorbidities* | 36 | 75 | 28 | 84.2 | 37.3 | 74.4 |

**Supplementary Table S1:** **Demographics of study cohort**. Groups separated according to cohort disease groupings and PASC status.


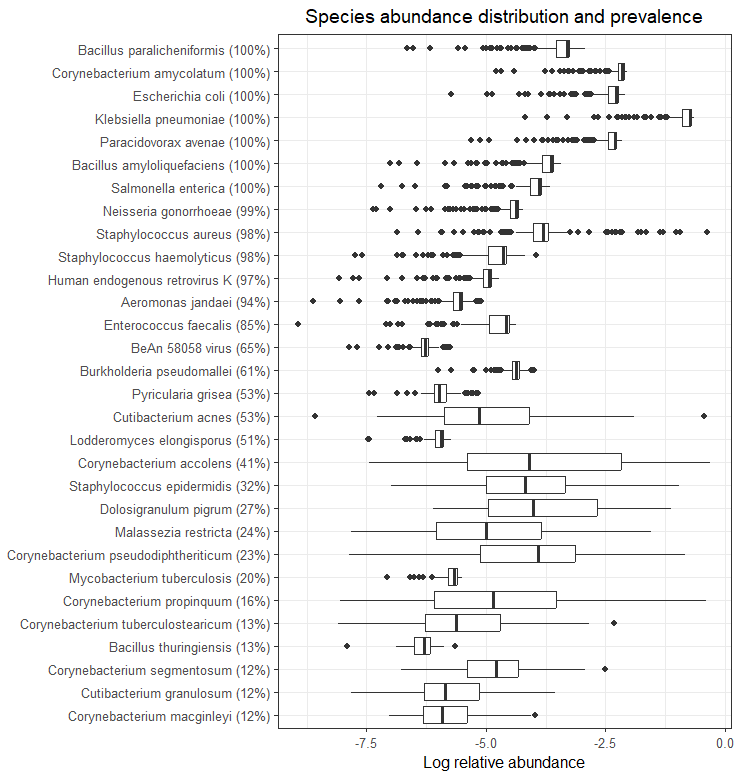


Supplementary figure S1: **Species abundance distribution and prevalence.** Boxplots of the log-transformed relative abundance for core microbial taxa (global prevalence >10%) are presented, with each taxon labelled and appended with its global prevalence percentage.

Supplementary figure S2: **Identified species from microbial community standards.** To assess the accuracy and reliability of the methodology, Zymo Microbial Community Standards (D6300) where included in each analytical batch from cell lysis to bioinformatic work, totalling 16 samples. **A** shows the abundance of species that were included in the community standard, with their true abundance in blue. **B** shows the identifications and abundances of species that were not included in the community standards (Split into correct Genus or incorrect Genus). For both graphs, relative abundance percentage is shown on the y-axis.


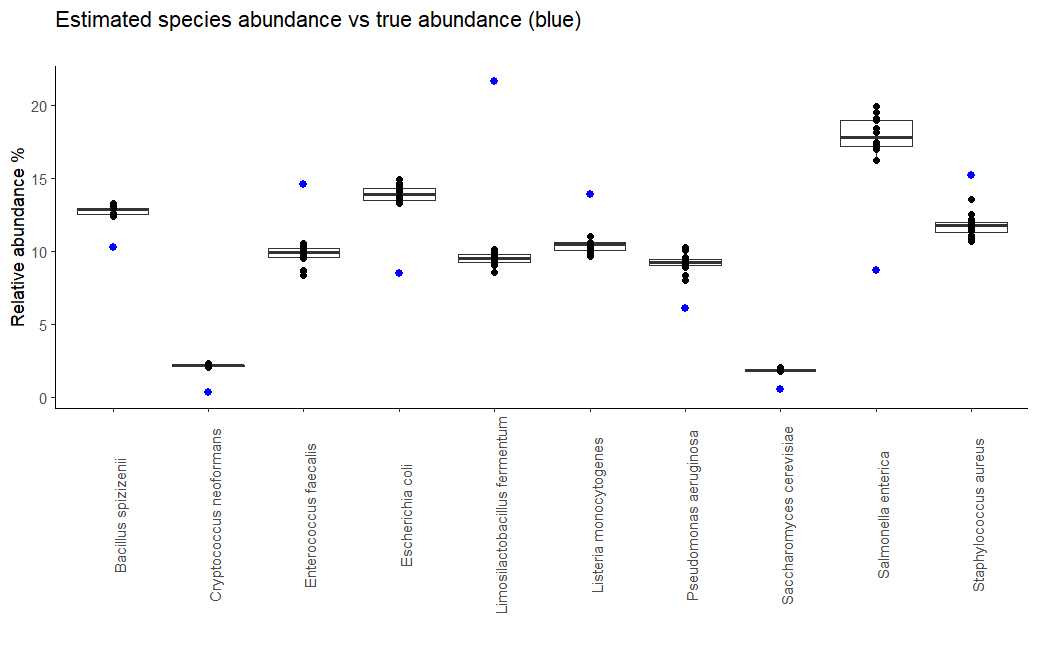

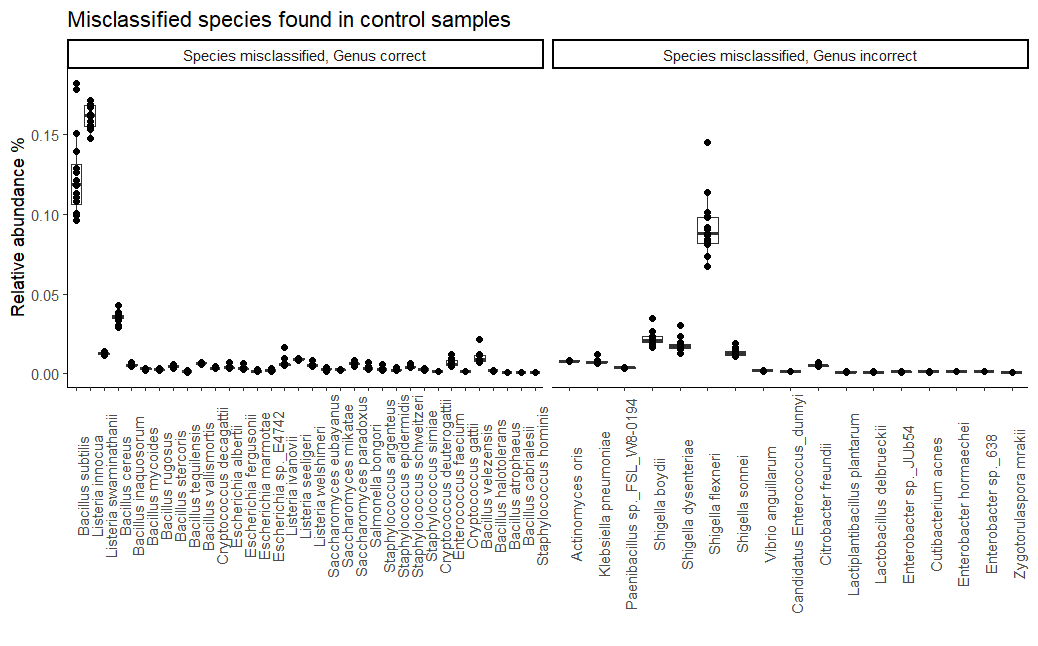


**A**

**B**
